# Defining the contribution of neuroinflammation to Parkinson’s disease in humanized immune system mice

**DOI:** 10.1186/s13024-017-0158-z

**Published:** 2017-02-14

**Authors:** Gunjan Dhawan Manocha, Angela Marie Floden, Kendra Lynn Puig, Kumi Nagamoto-Combs, Clemens R. Scherzer, Colin Kelly Combs

**Affiliations:** 1 Department of Biomedical Sciences, University of North Dakota School of Medicine and Health Sciences, Grand Forks, ND 58203 USA; 2 Department of Pathology, University of North Dakota School of Medicine and Health Sciences, Grand Forks, ND 58203 USA; 3 Neurogenomics Lab and Parkinson Personalized Medicine Initiative of Harvard Medical School and Brigham & Women’s Hospital, Cambridge, MA 02139 USA

**Keywords:** Parkinson’s disease, Humanized mice, MPTP, FK506, Neuroinflammation

## Abstract

**Background:**

Reactive microglia have been associated with the histological changes that occur in Parkinson’s disease brains and mouse models of the disease. Multiple studies from autopsy brains have verified the presence of microgliosis in several brain regions including substantia nigra, striatum, hippocampus and various cortical areas. MPTP injections in rodents have also shown striato-nigral microgliosis correlating with the loss of dopaminergic neurons. However, consistent data with respect to cytokine and immune cell changes during Parkinson’s disease have not been fully defined.

**Results:**

In order to improve understanding of the role of neuroinflammation in Parkinson’s disease, we employed the MPTP injection model using humanized CD34+ mice along with age-matched C57BL/6 mice. NSG mice engrafted with hu-CD34+ hematopoietic stem cells were injected with MPTP to quantify cytokine changes, neuron loss, gliosis, and behavioral dysfunction. The mice were also treated with or without the calcineurin/NFAT inhibitor, FK506, to determine whether modulating the immune response could attenuate disease. MPTP injections produced impairment of motor performance, increased microgliosis, elevated brain cytokine levels, and reduced tyrosine hydroxylase immunoreactivity in the substantia nigra and striatum of both humanized CD34+ mice and C57BL/6 mice with a strikingly different profile of human versus mouse cytokine elevations observed in each. Interestingly, FK506 injections significantly attenuated the MPTP-induced effects in the humanized CD34+ mice compared the C57BL/6 mice. In addition, analyses of human plasma from Parkinson’s disease donors compared to age-matched, healthy controls demonstrated an increase in a number of pro-inflammatory cytokines in female patients similar to that observed in MPTP-injected female CD34+ mice.

**Conclusions:**

This study demonstrates for the first time, induction of Parkinson’s disease-like symptoms in female humanized CD34+ mice using MPTP. The profile of cytokine changes in the serum and brains of the humanized CD34+ mice following MPTP injection differed significantly from that occurring in the more commonly used C57BL/6 strain of mice. Moreover, several cytokine elevations observed in the MPTP injected humanized CD34+ mice were similarly increased in plasma of PD patients suggesting that these mice offer the more relevant model for the inflammatory aspects of human disease. Consistent with this, the effects of MPTP on loss of tyrosine hydroxylase immunoreactivity, loss of motor strength, and increase in proinflammatory cytokines were attenuated using an immunosuppressant drug, FK506, in the humanized CD34+ but not the C57BL/6 mice. Collectively, these findings suggest that MPTP injected, humanized CD34+ mice represent a more accurate model for assessing inflammatory changes in PD.

## Background

Immunoreactive microglia have been reported in both human brains and animal models of Parkinson’s disease [1–7]. Multiple studies of autopsy brains have shown microgliosis in not just the substantia nigra but also the striatum, hippocampus and other cortical areas [5–13]. Reports using rodent models of disease also demonstrate increased microgliosis correlating with dopaminergic neuron loss using the rat 6-hydroxydopamine (6-OHDA) toxin injection model [3]. The LPS-injected rodent model of Parkinson’s disease (PD) also produces microgliosis [2]. Similarly, MPTP injection into rodents also results in striato-nigral microgliosis correlating with loss of dopaminergic cells [4]. Nevertheless, it is not yet clear whether the reactive microglia are a cause or effect of neuron loss during disease progression. Collectively, histologic findings from human brains and temporal analysis from rodent studies continue to support the idea that microglia activation and the associated inflammatory changes are part of the disease process.

In addition to the brain pathology, numerous studies report altered peripheral immune cell responses during disease suggesting a possible communication between the peripheral immune system and the central nervous system [14, 15]. A number of clinical studies have suggested that select inflammatory markers in the blood of PD patients may correlate with the degree of severity of the disease or disease-associated symptoms such as depression, anxiety, fatigue and sleep patterns changes [16–23].

In spite of the studies of human autopsy brains, clinical reports involving PD patients, and rodent models of the disease all having correlative findings, a number of conflicting reports exist. For example, animal models for PD have not been able to fully define the temporal changes of inflammatory response with respect to disease progression and neuron loss. Ideally, the rodent models of disease should accurately reflect the human condition to allow for mechanistic dissection. This is demonstrated concretely by the fact that only after characterization of human TNF-α overexpressing mice was TNF-α fully realized as a valid arthritis target [24].

In order to determine the contribution of neuroinflammatory changes to the neuron loss and motor dysfunction associated with PD, we employed a mouse humanized CD34+ line designed to contain engrafted multi-lineage human immune cell populations following injection of CD34+ hematopoietic stem cells [25]. The humanized CD34+ mice, along with age-matched C57BL/6 mice, were injected with MPTP to produce PD-like symptoms with or without intravenous injection of the immunosuppressive calcineurin/NFAT inhibitor drug, FK506, as a neuroprotectant. As expected, MPTP injections resulted in loss of dopaminergic neuron tyrosine hydroxylase (TH) immunoreactivity, decreased motor strength as seen from pole tests and grip strength tests, and increased microgliosis/astrogliosis. Interestingly, FK506 was able to attenuate the behavioral problems, cytokine levels, TH loss, and microgliosis/astrogliosis only in the humanized CD34+ mice. The mouse compared to human serum and brain cytokine profiles were also different across the two strains of mice. This study demonstrates for the first time that MPTP induces PD-like symptoms in female humanized CD34+ compared to female C57BL/6 mice. We also report a valid therapeutic target, calcineurin/NFAT activity, for treating motor impairment and inflammation in the humanized immune system model by using a clinically available drug, FK506. Finally, we validated that several of the peripheral cytokine changes observed in the MPTP-injected humanized CD34+ mice but not the C57BL/6 mice paralleled changes observed in human PD plasma suggesting that these mice may be a more accurate model for understanding human disease.

## Methods

### Animals

The humanized NOD scid gamma (NSG) mice and age-matched wild type mouse line, C57BL/6, were purchased from the Jackson Laboratory (Bar Harbor, Maine). For generation of humanized NSG mice, female NSG mice are injected with human hematopoietic stem cells (hCD34+) [25, 26]. Engraftment of mature human white blood cells (hCD45+) is confirmed 12 weeks after the injection [27]. Mice with more than 25% hCD45+ cells were considered successfully humanized and housed in a BSL2 negative pressure facility before shipping to UND. The mice obtained for this study were engrafted with hematopoietic stems cells from one individual with total hCD45+ cells engraftment ranging from 56 to 71%. The mice were 16 weeks of age at the time of arrival and allowed to acclimate for 7 days before experimentation. The mice were randomly divided into 4 groups: saline injection, MPTP injection, FK506 injection or MPTP and FK506 injection.

### Animal use

All animal use was approved by the University of North Dakota Institutional Animal Care and Use Committee (UND IACUC). Mice were provided food and water *ad libitum* and housed in a 12 h light/dark cycle. The investigation conforms to the National Research Council of the National Academies Guide for the Care and Use of Laboratory Animals (8th edition).

### Antibodies and reagents

Anti-TH antibody was purchased from EMD Millipore (Billerica, MA). Anti-Iba-1 antibody and anti-GFAP antibody were from Wako Chemicals (Richmond, VA) and Cell Signaling Technology, Inc. (Danvers, MA), respectively. The horseradish peroxidase conjugated secondary antibodies were purchased from Santa Cruz Biotechnology (Santa Cruz, CA). Mouse TNF-α ELISA kit was obtained from R&D Systems (Minneapolis, MN). Elite Vectastain ABC avidin and biotin kits, biotinylated anti-rabbit, anti-mouse, and anti-rat antibodies and the Vector VIP kits were from Vector Laboratories Inc. (Burlingame, CA). The anti-CD68 antibody was obtained from AbD Serotec (Raleigh, NC). Human specific anti-CD68 and anti-HLA-DR (LN3) antibodies were from Bio-Rad (Hercules, CA). The human specific anti-CD45 antibody was purchased from Dako (Carpinteria, CA).

### MPTP and FK506 treatments

The 16-week old female CD34+ mice and the age-matched C57BL/6 mice were given 3 intraperitoneal (i.p.) injections of saline vehicle or MPTP-HCl (18 mg/kg of free base) at 2 h intervals for a total of 3 injections. For FK506 treatments, mice were given saline vehicle or 10.0 mg/kg/day starting 30 min after the first MPTP injection and continuing through 4 additional days after the last MPTP injection, totaling 5 days of FK506 injections.

### Pole test

Following the MPTP and FK506 injections, mice were housed for an additional 3 days and behaviorally tested on day 8. Each animal was administered the pole test to assess locomotor activity as a measure of dopaminergic neuron function following the MPTP injections [28]. Briefly, mice were placed head-upward on the top of a vertical rough-surfaced pole (diameter 8 mm, height 55 cm) with a base that was positioned on a flat surface. The time until the mouse descended to the bottom of the pole/cage floor (locomotor activity time, TLA) was recorded with a maximum of 120 s. Mice were returned to their home cages after testing and the pole was wiped clean with 50% ethanol in between mice and allowed to dry before the next trial.

### Grip strength test via Kondziela’s inverted screen test

To test gross strength of the four limb muscles in mice, mice were challenged with the inverted screen test as previously described by Deacon *et al* [29]. A 43 x 43 cm^2^ square wire mesh frame was made for this purpose. The mesh was12 x 12 mm^2^ square formed by 1-mm diameter wires. The frame was 4 cm deep wooden beading to prevent mice from climbing to the other side of the mesh. After a 15 min rest following the pole test, each mouse was placed onto the center of the mesh square frame and the screen was rotated over the course of 2 s to an inverted position with the mouse head declining first. The screen was held at 40-50 cm over a clean surface, and the time it took each mouse to let go of the screen was measured until the maximum of 120 s was reached. After testing, mice were returned to their home cages.

### Tissue and serum collection

Following the behavioral tests, the mice were euthanized by i.p. injection of a mixture of 100 mg/kg ketamine/16 mg/kg xylazine, followed by cardiac exsanguination and PBS perfusion. Blood was collected and serum separated by centrifugation at 2000 x *g* for 10 min at 4 °C for ELISA arrays. The brain, spleen and intestines were collected from each mouse. The striatum was dissected out of the right cerebral hemisphere and flash frozen in liquid nitrogen. A portion of the ileum and spleen were also flash frozen. The left cerebral hemisphere and remaining spleen and ileum samples were immersion-fixed using 4% paraformaldehyde in PBS. The fixed tissue was cryoprotected through 2 successive 30% sucrose changes before sectioning.

### Human plasma and tissue

Age matched human plasma (Parkinson’s disease and healthy controls) were obtained from the Harvard Biomarkers Study (http://neurodiscovery.harvard.edu/biomarkers-core). Human normal adult spleen frozen sections were purchased from BioChain Institute, Inc. (Newark, CA). Slides were first processed for antigen retrieval by boiling in Tris-EDTA, pH 9.0, for 20 min, then immunostained along with mouse spleen and intestine sections as described below.

### Immunostaining mouse brains

The left cerebral hemispheres for humanized CD34+ and age-matched C57BL/6 mice (saline, FK506, MPTP and MPTP + FK506) were sectioned using a freezing microtome. Briefly, multiple paraformaldehyde-fixed and sucrose-equilibrated tissues were embedded in a 15% gelatin (in 0.1 M phosphate buffer, pH 7.4) matrix to form a sample block for simultaneous processing. The block was immersed in a 4% paraformaldehyde solution for 3-4 days to harden the gelatin matrix, followed by replacing the solution with 30% sucrose every 3-4 days each for 2 weeks. The blocks were then flash frozen using dry-ice/isomethylpentane, and 40 μm serial sections were cut using a freezing microtome. Serial sections (960 μm apart) were immunostained using anti-TH antibody (1:1000 dilution), anti-Iba-1 antibody (1:500 dilution) or anti-GFAP antibody (1:1000 dilution). The antigens were visualized using Vector ABC kit and Vector VIP as the chromogen (Vector Laboratories, Inc., Burlingame, CA) according to the manufacturer protocols. The slides were coverslipped using VectaMount (Vector Laboratories) following a standard dehydrating procedure through a series of ethanol concentrations and Histo-Clear (National Diagnostics, Atlanta, GA). Photomicrographs were taken using an upright Leica DM1000 microscope and Leica DF320 digital camera system. Quantitation of immunostaining was performed as previously described [30]. Briefly, optical densities from the striatum, substantia nigra, and hippocampus regions from the same serial sections were measured using Adobe Photoshop software (Adobe Systems, San Jose, CA). The values for each section were averaged (4 sections/brain, 4-5 brains per condition) and compared.

### Immunostaining peripheral tissue

Spleen and ileum samples were serially cut (10 μm) onto gelatin subbed slides using a cryostat. These sections, along with human spleen slides (positive controls), were immunostained using anti-CD68 (1:500), anti-hCD68 (1:500), anti-HLA-DR (LN3) (1:500) and anti-hCD45 (1:250) antibodies. The immunostaining was visualized using Vector VIP as the chromogen.

### ELISA arrays

Striatal samples and serum from all groups of humanized CD34+ and C57BL/6 mice were flash frozen following collection. Pre-weighed samples from mice and human plasma samples were used for multi-analyte ELISA arrays (Qiagen, Valencia, CA) and the levels of various cytokines were determined as per the manufacturer protocol. Human and mouse Inflammatory Cytokines Multi-Analyte ELISArray kits from Qiagen were chosen in order to obtain expression of a wide array of inflammatory and anti-inflammatory cytokines. Cytokine concentrations were normalized by tissue weight.

### Design-based stereological quantification of Iba-1 positive cells

Paraformaldehyde-imbedded sections from humanized CD34+ and age-matched C57BL/6 mice (saline, FK506, MPTP and MPTP + FK506) were cut at 40 μm intervals and immunolabled for Tyrosine Hydroxylase (TH), Iba-1 and GFAP. Sections from three different mice for each condition of mice were assessed for TH, GFAP or Iba-1-positive nuclei with the section interval set at 960 μm. Non-biased quantification of immuno-positive nuclei was conducted using the optical fractionator method originally developed by West and colleagues [31] and adapted from previous studies with other neural populations [32, 33]. TH, GFAP or Iba-1-positive cells in the temporal cortex layer were counted in every 24th section using StereoInvestigator 11.06.2 (Microbrightfield Inc., Williston, VT) on an Olympus BX51WI equipped with a motorized x, y, and z stage. In each animal, immuno-positive cells were counted from 21 to 30 randomly and systematically selected frames in every serial section using the 40x objective. For cell counting, the contour of the Substantia Nigra was delineated with 2× magnification based on standard anatomical markers and the grid size set following sub-sampling optimization. In brief, the number and location of counting frames and the counting depth for that section were determined by entering parameters for the grid size (300 X 300 μm), the thickness of top guard zone (2 μm) and the optical dissector height (26 μm). The guard zones were set at 2 μm above and below the counting depth for each section with regional thickness and variation in section integrity taken into consideration and the max and min z-depth determined at each sampling location prior to counting. The optimal counting frame width (100 μm) and height (100 μm) were determined with an initial oversampling of a representative set of sections from control and treated samples. The cell bodies of the TH, GFAP or Iba-1-positive cells were counted if they were entirely within the 26 μm depth of the counting frame and only at the z-depth at which the nucleus was in focus. The StereoInvestigator software calculated the total number of Iba-1, TH, and GFAP-positive cells in the temporal cortex using the 3D optical fractionator formula designated: *N =* t/h X 1/ssf X 1/asf X 1/hsf X ΣQ-. For the calculations, “t/h” is the section mounted thickness over the counting frame height; “ssf” is the section sampling fraction; “asf” is the area sampling fraction, which was calculated by dividing the area sampled with total area of the temporal cortex (in our tracings, the sum of temporal cortex areas sampled in every 24th section); “hsf” is the height sampling fraction, which was determined by dividing the height of the counting frame (26 μm) by the section thickness determined for each sampling site (37-39 μm on average); and “ΣQ”- denotes the total count of particles sampled for the entire temporal cortex. The sampling was optimized for maximal efficiency, with a final mean coefficient of error (CE, Gundersen) of less than 10% for all sections sampled. One-way analysis of variance (ANOVA; GraphPad Prism, 6.0c; LaJolla, CA) was used to analyze positive nuclei and all data are presented as mean ± SD for *n =* 3 samples.

### Statistical analysis

Data are presented as mean ± standard deviation. Values statistically different from controls were determined using one-way ANOVA (or two-way ANOVA where required). The Turkey-Kramer multiple comparisons post-test was used to determine p values.

### Statistical analysis for human plasma cytokine ELISA arrays

A total of 140 plasma samples were analyzed to determine cytokine level differences in 4 groups: female Parkinson’s disease donors, female healthy controls, male Parkinson’s disease donors and male healthy controls. Initial sample size for all 4 conditions was *n =* 35. Following ELISA experiments for 12 cytokines, the samples that were below the detection limit were excluded from the analyses. The samples that did not represent the population for a 95% confidence interval i.e. the values that were 2 times standard deviations above or below the mean were also excluded from the analyses. This resulted in a sample size ranging from 14 to 34 for all the cytokines but IL-4 (*n =* 5-7). Values statistically different between the groups were determined using two-way ANOVA with Holm-Sidak post-hoc test.

## Results

### MPTP injected C57BL/6 and humanized CD34+ mice showed reduced motor strength based on the grip strength and pole tests

**Fig. 1:**
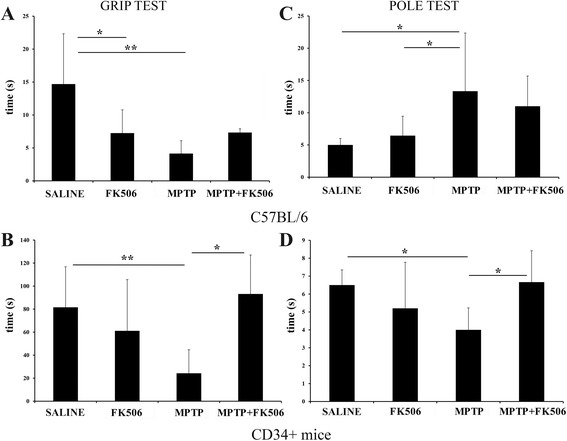
MPTP injected C57BL/6 and humanized CD34+ mice showed reduced motor strength based on the grip strength and pole tests. The hCD34+ mice and age-matched C57BL/6 female mice were intraperitoneal injected three times with saline vehicle or MPTP-HCL (18 mg/kg) at 2 h intervals followed by FK506 injections (10 mg/kg/day) for 5 days. Mice were tested for grip strength (**a**, **b**) and pole test (**c**, **d**) 7 days post MPTP injections. Results were averaged from 8 to 10 mice (CD34+) and 4-10 (C57BL/6) per group and plotted ± SD (*p < 0.05)

Following MPTP and/or FK506 injections, the motor function of mice was assessed using the grip strength and pole tests. As expected, the MPTP injection groups of both humanized CD34+ and C57BL/6 mice showed deteriorated performance in both tests (Fig. 1). Interestingly, FK506 significantly improved both pole test and grip strength performance of the MPTP injected humanized CD34+ mice (Fig. 1, b & d). However, FK506 treatment of MPTP-injected C57BL/6 mice failed to improve performance in either of the tests (Fig. 1, a & c). The difference between general time values for the CD34+ and C57BL/6 strains in grip strength can be attributed to our observations that C57BL/6 mice were unable to hold onto the test surface (inverted mesh frame) for very long irrespective of the treatment group they belonged to. Humanized CD34+ mice, in general, held onto the mesh surface much longer, hence their time values on the y-axis are quite different even in the saline-injected mice compared to C57BL/6 mice.

### FK506 attenuated MPTP-mediated decrease in TH immunoreactivity in the striatum and substantia nigra of humanized CD34+ mice but not C57BL/6 mice

**Fig. 2:**
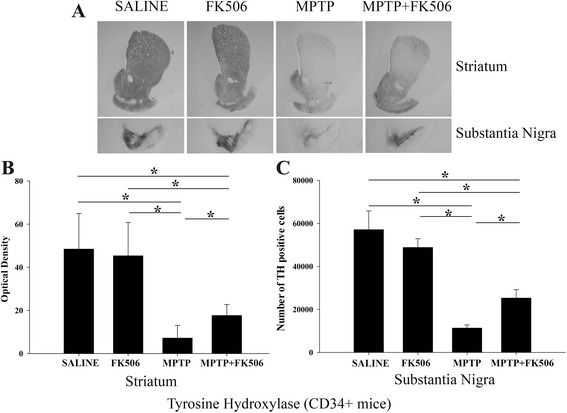
FK506 attenuated the decrease of TH immunoreactivity in the striatum and substantia nigra of MPTP injected humanized CD34+ mice. The hCD34+ mice female mice were intraperitoneal injected three times with saline vehicle or MPTP-HCL (18 mg/kg) at 2 h intervals followed by FK506 injections (10 mg/kg/day) for 5 days. Eight days post MPTP injections, brains were dissected out and right hemispheres fixed and immunostained using anti-TH antibody. **a** Representative images from striatum and substantia nigra are shown at 1× magnification. **b** Optical density of immunopositive staining from striatum and **c** number of TH-positive cells from substantia nigra were measured and averaged 4-5 sections per brain and 4–5 animals per group ± SD (*p < 0.05)

**Fig. 3:**
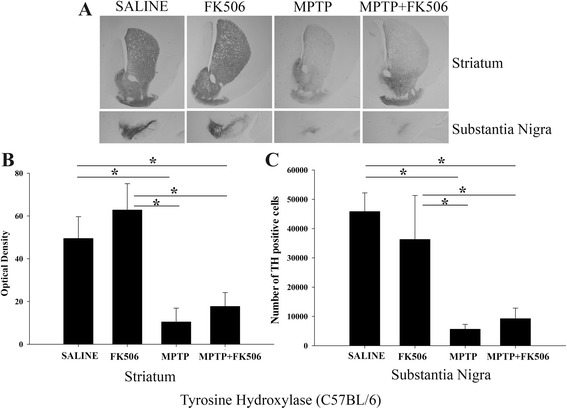
FK506 provided no protection of TH immunoreactivity in the striatum and substantia nigra of MPTP injected C57BL/6 mice. C57BL/6 female mice were intraperitoneal injected three times with saline vehicle or MPTP-HCL (18 mg/kg) at 2 h intervals followed by FK506 injections (10 mg/kg/day) for 5 days. Eight days post MPTP injections, brains were dissected out and right hemispheres fixed and immunostained using anti-TH antibody. **a** Representative images from striatum and substantia nigra are shown at 1× magnification. **b** Optical density of immunopositive staining from striatum and **c** number of TH-positive cells from substantia nigra were measured and averaged for 4–5 sections per brain from 4 to 5 animals per group ± SD (*p < 0.05)

To assess the effect of MPTP on dopaminergic neurons, brain sections from all groups of animals were immunostained using anti-TH antibody as the dopaminergic cell marker to quantify changes via both densitometric and stereologic evaluation. Tyrosine hydroxylase (TH) is the rate limiting enzyme in the synthesis of dopamine. Therefore, it is a reliable marker for neuronal dopamine synthesis. As expected, MPTP injections reduced TH immunoreactivity in both the substantia nigra and striatum of the humanized CD34+ mice (Fig. 2a, “MPTP”). In contrast, FK506 treatment significantly attenuated the MPTP-mediated reduction in TH immunoreactivity (Fig. 2a, MPTP + FK506). Quantitation of the immunoreactivity indicated that the MPTP/FK506 group had approximately 2-fold more TH immunoreactivity in the striatum and a similar increase in TH positive cell numbers in the substantia nigra when compared to the MPTP only group (Fig. 2, b &c). To compare the effects of FK506 in C57BL/6 mice, TH immunoreactivity was again quantified from striatum and substantia nigra using densitometric and stereologic evaluation. MPTP-injected mice had decreased TH immunoreactivity in both regions as was observed with the humanized CD34+ mice (Fig. 3). However, unlike humanized CD34+ mice, FK506 treatment did not significantly affect the MPTP-dependent decrease in TH immunoreactivity in the C57BL/6 mice in either region (Fig. 3, b &c). However, it should be noted that only 4 out of 10 treated animals survived from the MPTP + FK506 treatment suggesting that the combination of drugs was more toxic to the C57BL/6 females.

### MPTP injections increased microgliosis in the striatum, substantia nigra, and hippocampus of humanized CD34+ mice

**Fig. 4:**
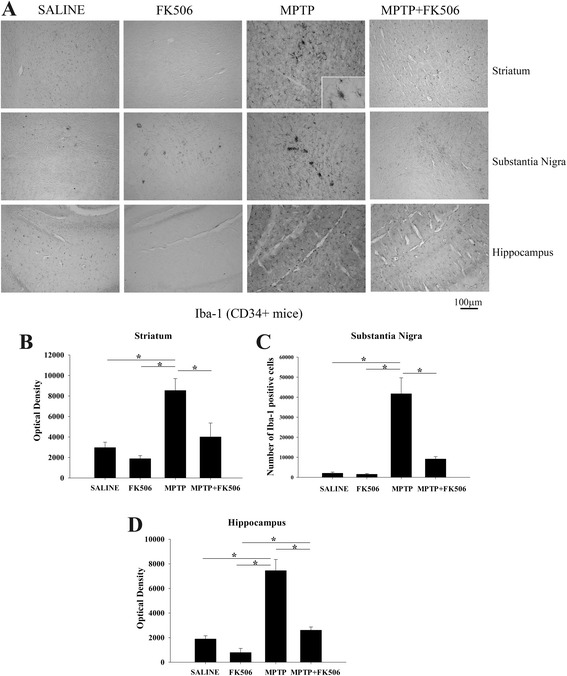
MPTP injections increased microgliosis in the striatum, substantia nigra, and hippocampus of humanized CD34+ mice. The hCD34+ mice were intraperitoneal injected three times with saline vehicle or MPTP-HCL (18 mg/kg) at 2 h intervals followed by FK506 injections (10 mg/kg/day) for 5 days. Eight days post MPTP injections, brains were dissected out and right hemispheres fixed and immunostained using anti-Iba-1 antibody (microglia marker). **a** Representative images from striatum, substantia nigra and hippocampus are shown at 10× magnification with 63× magnification insert. Optical density of immunopositive staining from **b** striatum and **d** hippocampus were measured from 3 to 4 optical fields at 10× magnification per section from 4 to 5 animals per group and **c** number of Iba-1 positive cells from the substantia nigra were counted and averaged ± SD (*p < 0.05)

To examine microglial activation following MPTP injections, brain sections from MPTP- and FK506-injected humanized CD34+ mice were immunostained for a microglia marker, Iba-1. The staining intensity and the morphology of the stained cells were then analyzed to determine the activation status of microglia. The mice injected with MPTP showed increased microgliosis in the striatum, substantia nigra and hippocampus as compared to saline- and FK506-injected mice, while concomitant FK506 injections visibly attenuated the MPTP-induced microgliosis (Fig. 4a). Densitometric quantitation of Iba-1 immunoreactivity from the striatum and hippocampus and stereological quantitation of Iba-1 positive cells from substantia nigra indicated that FK506 treatment significantly decreased MPTP-induced microgliosis (Fig. 4, b-d). This demonstrated that the behavioral improvements and neuroprotection provided by FK506 in these mice correlated with decreased microglial activation.

### MPTP injections increased microgliosis in the striatum, substantia nigra, and hippocampus of C57BL/6 mice

**Fig. 5:**
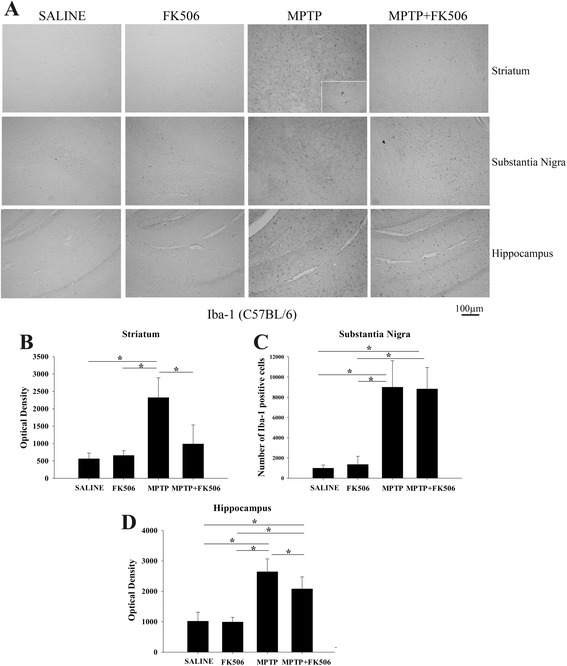
MPTP injections increased microgliosis in the striatum, substantia nigra, and hippocampus of C57BL/6 mice. C57BL/6 female mice were intraperitoneal injected three times with saline vehicle or MPTP-HCL (18 mg/kg) at 2 h intervals followed by FK506 injections (10 mg/kg/day) for 5 days. Eight days post MPTP injections, brains were dissected out and right hemispheres fixed and immunostained using anti-Iba-1 antibody (microglia marker). **a** Representative images from striatum, substantia nigra and hippocampus are shown at 10× magnification with 63× magnification insert. Optical density of immunopositive staining from **b** striatum and **d** hippocampus were measured for 3–4 optical fields at 10× magnification per section from 4 to 5 animals per group and **c** number of Iba-1 positive cells from substantia nigra were counted and averaged ± SD (*p < 0.05)

For comparison purposes, microgliosis in MPTP-injected C57BL/6 mice was also quantified. The C57BL/6 mice injected with MPTP showed significantly higher Iba-1 immunoreactivity in the striatum, substantia nigra, and hippocampus as was observed in the humanized CD34+ mice. FK506 treatment also reduced the MPTP-induced microgliosis in the striatum and hippocampus (Fig. 5, a&b). However, unlike the humanized CD34+ mice, the effect of FK506 in the substantia nigra was not significant in C57BL/6 mice (Fig. 5c). This demonstrated that in spite of no behavioral recovery or neuroprotection in these mice, the drug had some modest effect on microglial activation.

### MPTP injections increased astrogliosis in the substantia nigra and hippocampus of humanized CD34+ mice

**Fig. 6:**
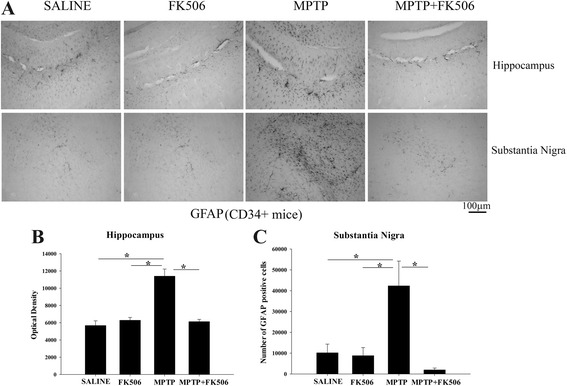
MPTP injections increased astrogliosis in the substantia nigra and hippocampus of humanized CD34+ mice. The hCD34+ mice were intraperitoneal injected three times with saline vehicle or MPTP-HCL (18 mg/kg) at 2 h intervals followed by FK506 injections (10 mg/kg/day) for 5 days. Eight days post MPTP injections, brains were dissected out and right hemispheres fixed and immunostained using anti-GFAP antibody (astrocyte marker). **a** Representative images from substantia nigra and hippocampus are shown at 10× magnification. Optical density of immunopositive staining from the **b** hippocampus was measured and averaged for 3–4 optical fields at 10× magnification per section from 4–5 animals per group and **c** number for GFAP positive cells from the substantia nigra were counted and averaged ± SD (*p < 0.05)

MPTP has been shown to also induce astrocyte activation in mice [34]. In order to examine astroglial activation in the MPTP-injected humanized CD34+ mice, brain sections were immunostained for glia fibrillary acidic protein (GFAP), an astrocyte-specific marker. MPTP induced marked astrocyte activation both in the substantia nigra and hippocampus (Fig. 6) while no GFAP immunostaining was observed in the striatum of these mice (data not shown). Similar to microgliosis, MPTP-induced astrogliosis was suppressed by FK506 in both regions (Fig. 6a). Densitometric analysis showed that MPTP increased GFAP immunoreactivity by approximately 2-fold in the hippocampus while the number of GFAP positive cells in the substantia nigra showed a 4-fold increase with MPTP, and FK506 significantly and almost entirely reduced the MPTP-mediated increase in GFAP immunoreactivity (Fig. 6, b & c).

### MPTP injections increased astrogliosis in the substantia nigra of C57BL/6 mice

**Fig. 7:**
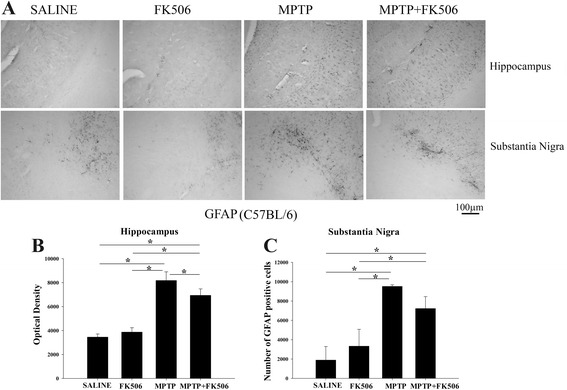
MPTP injections increased astrogliosis in the hippocampus of C57BL/6 mice. C57BL/6 female mice were intraperitoneal injected three times with saline vehicle or MPTP-HCL (18 mg/kg) at 2 h intervals followed by FK506 injections (10 mg/kg/day) for 5 days. Eight days post MPTP injections, brains were dissected out and right hemispheres fixed and immunostained using anti-GFAP antibody (astrocyte marker). **a** Representative images from substantia nigra and hippocampus are shown at 10× magnification. Optical densities of immunopositive staining from the **b** hippocampus was measured and averaged for 3–4 optical fields at 10× magnification per section from 4 to 5 animals per group and **c** number for GFAP positive cells from the substantia nigra were counted and averaged ± SD (*p < 0.05)

Astrogliosis in the MPTP-injected C57BL/6 mice was also quantified in parallel with the humanized CD34+ mice. The MPTP-injected mice showed a significantly higher level of GFAP immunoreactivity in the hippocampus as was observed in the humanized CD34+ mice (Fig. 7a). However, FK506 treatment reduced the hippocampal MPTP-mediated astrogliosis only marginally (Fig. 7b). Furthermore, MPTP injections resulted in a significant increase in GFAP stained cells in the substantia nigra in C57BL/6 mice (Fig. 7c). Similarly, the effect of FK506 on inhibition of substantia nigra astrogliosis in the MPTP-injected C57BL/6 mice was not statistically significant. There were no GFAP immuno-positive cells in the striatum of these mice as in the humanized CD34+ mice (data not shown).

### Human HLA-DR and CD45 immunoreactivity was observed in the spleen, intestine and brain of the humanized CD34+ mice

**Fig. 8:**
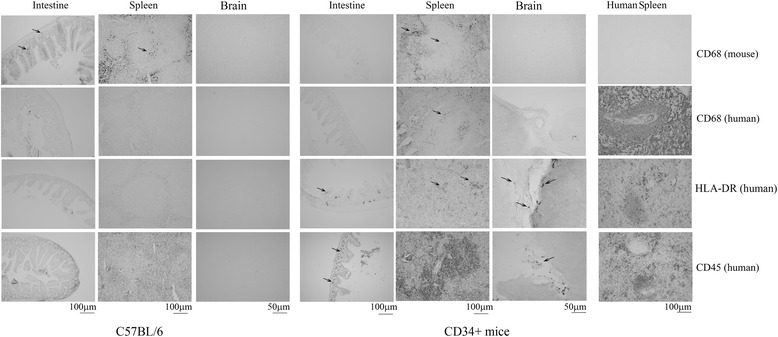
Human HLA-DR and human CD45 immunoreactivities were observed in the spleens, intestines, and occasionally in brain meninges of the humanized CD34+ mice. Intestines, spleens and brains (right hemispheres) were dissected out from 16 weeks old, female C57BL/6 and hCD34+ MPTP injected mice and fixed using 4% paraformaldehyde. Fixed tissue along with human spleen sections (positive controls) were immunostained using anti-CD68 (rodent specific), anti-CD68 (human specific), anti-HLA-DR (LN3, human specific), and anti-CD45 (human specific) antibodies. Intestine and spleen representative images at 10× magnification and brain representative images at 20× magnification are shown

To determine whether human macrophages in the humanized CD34+ mice contributed to the changes observed in the spleen, intestine, and brain by infiltrating into these regions, macrophage proteins, CD68, CD45 and HLA-DR (LN3) were immunodetected using human-specific antibodies. Mouse-specific CD68 antibody was also used to differentiate macrophages/microglia of mouse origin. Sparse, but clearly detectable immunoreactivity for human HLA-DR and CD45 were found in the intestines of the CD34+ mice (Fig. 8). Furthermore, the spleens from these animals also showed robust immunoreactivity for human CD68 in addition to HLA-DR and CD45. However, the brain sections from these mice exhibited only minimal immunoreactivity for the human antigens. Human HLA-DR and human CD45 immunoreactivity was occasionally observed in the meningeal layers (Fig. 8), suggesting that human macrophages have limited infiltration into the brain. There was no CD68 immunoreactivity observed in the brain consistent with the lack of CD68 immunostaining in the intestines (Fig. 8). To test the specificity of the human-specific antibodies, spleens and intestines from C57BL/6 mice were immunostained as negative controls. As expected, the human-specific antibodies showed no immunoreactivity for CD68, CD45 and HLA-DR in these mice, which produced only mouse antigens. As a positive control, human spleen sections were immunostained using the human-specific CD68, CD45 and HLA-DR antibodies (Fig. 8, right column). All of these antibodies showed positive immunoreactivity in these sections, indicating their specificity to the human macrophage marker proteins.

### FK506 downregulated the MPTP-induced increase of serum inflammatory cytokine levels in the humanized CD34+ mice

**Fig. 9:**
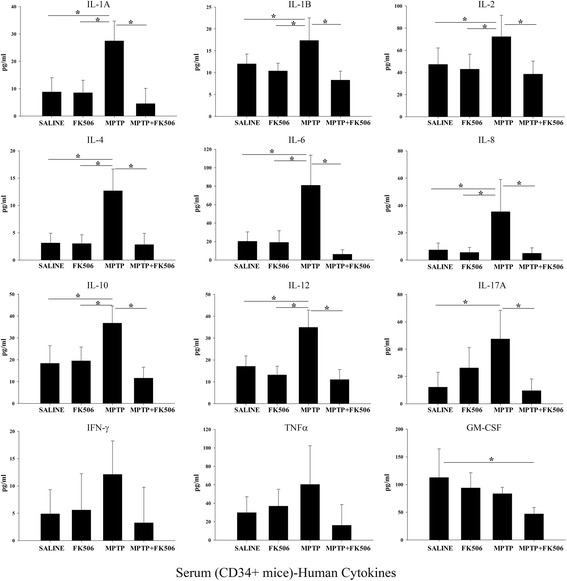
FK506 attenuated the MPTP-dependent increase of human inflammatory cytokine levels in humanized CD34+ mice serum. The hCD34+ mice were intraperitoneal injected three times with saline vehicle or MPTP-HCL (18 mg/kg) at 2 h intervals followed by FK506 injections (10 mg/kg/day) for 5 days. Eight days post MPTP injections, serum was collected and used for human specific multi-analyte cytokine ELISA arrays. Cytokine levels were determined from 6 animals per group ± SD (*p < 0.05)

Based on the findings that MPTP induced microgliosis in the brain and subsequent FK506 treatment reduced it in the humanized CD34+ mice, it was likely that inflammatory cytokine levels were elevated by MPTP and downregulated by FK505. Since few human cells were detected in the brains of CD34+ mice, we hypothesized that MPTP-induced microgliosis in the brain occurred primarily via cross-talk between peripheral cytokines and brain microglia. However, since HLA-DR immunoreactivity was observed in the brain tissue, though sparse, we cannot rule out the possibility of parenchymal cytokine production in the brains of these mice. In order to test the idea of periphery to brain communication, we next determined the serum inflammatory cytokine profile in these mice using human-specific multi-analyte ELISA arrays. MPTP-injected humanized CD34+ mice showed increased levels of IL-1α, IL-1β, IL-2, IL-4, IL-6, IL-8, IL-10, IL-12 and IL-17A cytokines (Fig. 9). As anticipated, all of the above cytokines were significantly downregulated by the concomitant FK506 treatment. The levels of IFN-γ, TNFα and GM-CSF were not affected by either MPTP or subsequent FK506 treatment.

### Serum of humanized CD34+ and C57BL/6 mice displayed differential changes in levels of mouse cytokines

**Fig. 10:**
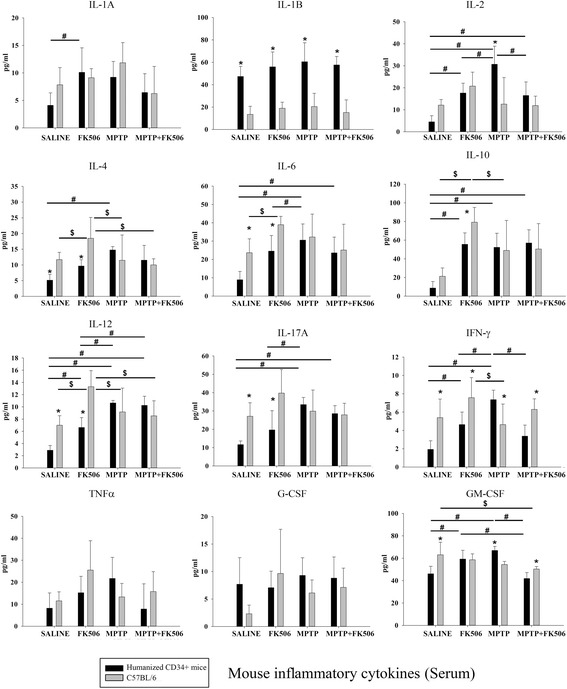
Serum of humanized CD34+ and C57BL/6 mice displayed differential changes in levels of mouse cytokines. Humanized CD34+ and C57BL/6 female mice were intraperitoneal injected three times with saline vehicle or MPTP-HCL (18 mg/kg) at 2 h intervals followed by FK506 injections (10 mg/kg/day) for 5 days. Eight days post MPTP injections, serum was collected and used for mouse specific multi-analyte cytokine ELISA arrays. Cytokine levels were determined from 4 to 6 animals per group ± SD (*p < 0.05 hCD34+ vs. C57BL/6; #*p <* 0.05 vs. hCD34+ mice, $*p <* 0.05 vs. C57BL/6)

We next examined the serum levels of mouse-specific cytokines in the humanized CD34+ and C57BL/6 mice for comparison across the strains. Mouse-specific inflammatory cytokine ELISA arrays indicated that the cytokine responses to MPTP administration in humanized CD34+ mice were distinct from those of C57BL/6 mice (Fig. 10). MPTP increased the serum levels of mouse cytokines, IL-2, IL-4, IL-6, IL-10, IL-12, IL-17A, IFN-γ and GM-CSF, more notably in the CD34+ mice. MPTP-induced changes in mouse versus human cytokine profiles were also compared in the humanized CD34+ mice. In contrast to the effect on human cytokine levels, FK506 treatment only attenuated levels of the mouse cytokines, IL-2, IFN-γ and GM-CSF. Unlike the humanized mice, MPTP injections in C57BL/6 mice did not affect mouse cytokine levels in serum (Fig. 10).

### MPTP injections increased levels of human inflammatory cytokines in the striatum of humanized CD34+ mice

**Fig. 11:**
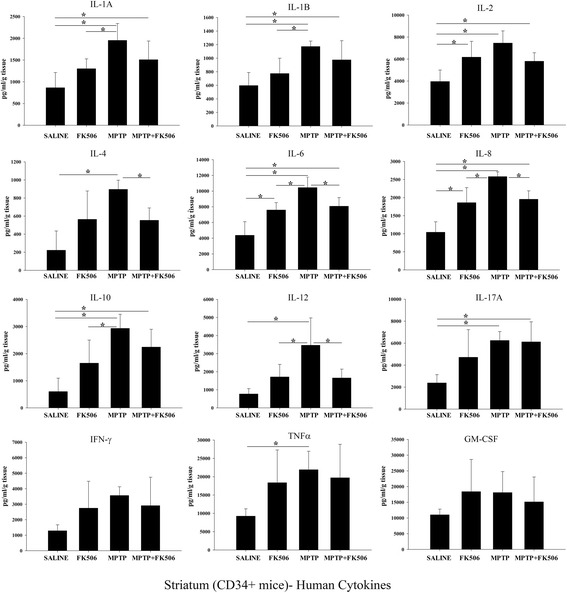
MPTP injections increased levels of human inflammatory cytokines in the striatum of humanized CD34+ mice. The hCD34+ mice were intraperitoneal injected three times with saline vehicle or MPTP-HCL (18 mg/kg) at 2 h intervals followed by FK506 injections (10 mg/kg/day) for 5 days. Eight days post MPTP injections, striatum were dissected out, lysed and used for human specific multi-analyte cytokine ELISA arrays. Cytokine levels were determined from 6 animals per group ± SD (*p < 0.05)

Since MPTP injections increased inflammatory cytokine levels in the serum of humanized CD34+ mice we examined whether the inflammatory response was translated to the brain. MPTP injected humanized CD34+ mice had increased levels of human IL-1α, IL-1β, IL-2, IL-4, IL-6, IL-8, IL-10 and IL-12, IL-17A and TNF-α compared to saline injected mice (Fig. 11). Based upon that fact that there were few detectable macrophages in the brain (Fig. 6), this data suggests that the elevated levels of human cytokines may be transported in from the blood. Interestingly, FK506 treatment only significantly decreased cytokine levels of IL-4, IL-6, IL-8 and IL-12 in the striatum following MPTP injections (Fig. 11).

### The striatum of humanized CD34+ and C57BL/6 mice displayed different murine cytokine levels after MPTP or FK506 injection

**Fig. 12:**
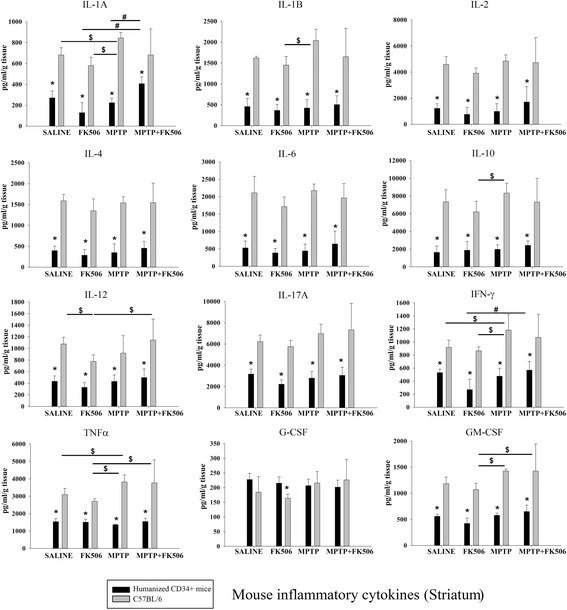
The striatums of humanized CD34+ and C57BL/6 mice displayed different murine cytokine levels after MPTP or FK506 injection. Humanized CD34+ and C57BL/6 female mice were intraperitoneal injected three times with saline vehicle or MPTP-HCL (18 mg/kg) at 2 h intervals followed by FK506 injections (10 mg/kg/day) for 5 days. Eight days post MPTP injections, striatum was dissected out, lysed and used for mouse specific multi-analyte cytokine ELISA arrays. Cytokine levels were determined from 4 to 6 animals per group ± SD (*p < 0.05 hCD34+ vs. C57BL/6; #*p <* 0.05 vs. hCD34+ mice, $*p <* 0.05 vs. C57BL/6 mice)

As with the serum, we next compared mouse brain cytokine differences across strains and for comparison to human brain cytokine levels in the humanized CD34+ mice. Overall, as expected, mouse cytokines levels were significantly lower in striatum of humanized CD34+ as compared to C57BL/6 mice (Fig. 10). There were no significant differences in striatal cytokine levels between saline and MPTP injected humanized CD34+ mice (Fig. 10). This demonstrated a clear difference between human and mouse cytokine changes in the brains of the MPTP injected humanized CD34+ mice. On the other hand, compared to saline injected animals the MPTP injected C57BL/6 mice had higher striatal IL-1α, IL-1β, IFN-γ and TNFα levels (Fig. 12). FK506 treatment had no effect on the MPTP-induced increase consistent with no behavioral or histologic improvement from drug treatment in the C57BL/6 female mice.

### Human PD plasma had elevated levels of IL-1α, IL-2, IL-4 and IL-6 compared to healthy controls

**Fig. 13:**
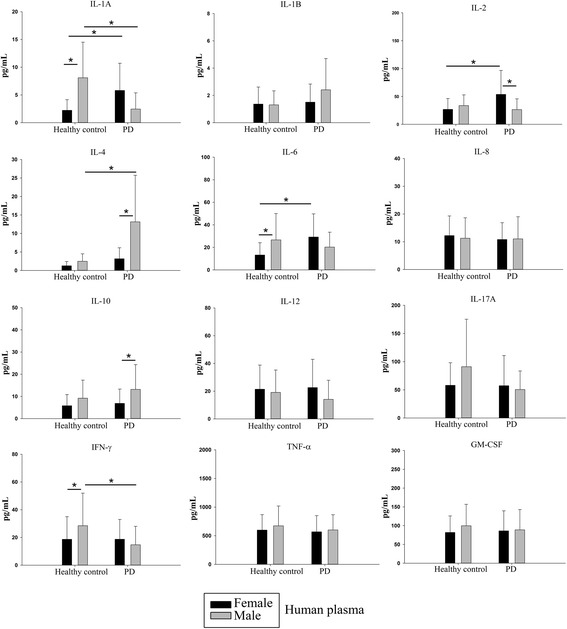
Human PD plasma had elevated levels of IL-1α, IL-2, IL-4 and IL-6 compared to healthy controls. Plasma from male and female Parkinson’s disease donors and age-matched healthy controls was used to perform cytokine ELISAs. Mean values from 14 to 34 patients per condition were averaged and graphed ± SD (*p < 0.05)

In order to validate that the inflammatory changes in CD34+ humanized mice represented the human disease, human plasma cytokine levels were quantified via ELISA from Parkinson’s disease donors and healthy controls. Female patients with Parkinson’s disease (PD) showed elevated levels of IL-1α, IL-2 and IL-6 while male patients with PD showed elevated levels of only IL-4 compared to healthy controls (Fig. 13). Interestingly, IFN-γ levels in male patients with PD were significantly lower than in male controls. Although there were significant differences in multiple cytokine levels between males and females (Fig. 13), the increase in some of the inflammatory cytokines in plasma of female patients with PD compared to age-matched female controls paralleled the observed increases in serum and brains of the female MPTP-injected humanized CD34+ mice. This data is largely consistent with a previously reported study by Nagatsu et al. demonstrating an increase in various cytokines (TNF-α, IL-1β, IL-2, IL-4 and IL-6 in the striatum and cerebrospinal fluid of Parkinson’s disease patients [35].

## Discussion

Our findings suggest that female C57BL/6 and female humanized CD34+ mice can both be used with the MPTP model of PD. Moreover, we observed profound strain as well as species differences with respect to MPTP-induced changes and their response to anti-inflammatory FK506 treatment. MPTP injection produced the expected impairment of motor performance, increased microgliosis, increased astrogliosis, elevated brain cytokine levels, and reduced TH immunoreactivity in the substantia nigra and striatum of C57BL/6 mice. However, there were no changes in serum cytokine levels in the female C57BL/6 mice following MPTP treatment and FK506 provided no significant protective effects as assessed by behavior, TH immunoreactivity, Iba-1 microglial immunoreactivity, or brain and serum cytokine levels. This suggests, for reasons unclear, that the drug did not have a robust anti-inflammatory effect in the C57BL/6 mice. A similar decrease in TH immunoreactivity, increased microgliosis, astrogliosis and impaired motor performance was observed in the MPTP injected humanized CD34+ mice demonstrating conservation of toxin-mediated neuron death and disease modeling in this line. In addition, these mice had an increase in select mouse cytokines in the both blood and brains following MPTP injection quite different from changes observed in the C57BL/6 mice. More importantly, both blood and brain levels of specific human cytokines were elevated in the MPTP injected mice demonstrating a possibility that the blood cytokine level profile may be used as a biomarker of brain cytokine levels and neuronal loss. This idea is further supported by cytokine analyses of human PD plasma compared to healthy controls. Plasma from female PD patients and the MPTP-injected humanized CD34+ mice showed an increase in IL-1α, IL-2, and IL-6 compared to controls. This suggests that these cytokines could be further validated as potential biomarkers for the disease, possibly in a sex selective fashion. In addition, it would be very interesting to further study the direct effects of these cytokines on brain changes in the humanized CD34+ mice. Finally, unlike the C57BL/6 findings, FK506 treatment had a dramatic effect on improving behavioral performance, increasing TH immunoreactivity, decreasing microglial Iba-1 immunoreactivity, decreasing GFAP astroglial immunoreactivity, and lowering serum cytokine levels in the CD34+ humanized mice suggesting potential for human translation. These data suggest that the humanized immune system mice provide a unique resource for characterizing the contribution of inflammatory changes to disease.

Human CD34+ hematopoietic stem-cell engrafted NSG mice develop multi-lineage human immune cells and have become a popular, valid platform for infectious disease research and cancer biology, especially with respect to immune-oncology studies [36]. Engraftment of human hematopoietic stem cells is much more efficient in female scid mice as compared to males [37]. Therefore humanized CD34+ mice from Jackson Laboratories can only be obtained as females. In this study, age-matched female C57BL/6 mice were used for comparing differences between the human and mouse systems. Many murine MPTP-related studies are typically performed on male mice to avoid the increased risk of fatalities observed with females [38]. However, even more surprising to us was the fact that the combined MPTP + FK506 treatment group experienced higher levels of toxicity in the C57BL/6 strain totaling near 50% of animal loss in this group and not the MPTP or FK506 alone mice. In addition, with the limited numbers surviving in this group there were no drug-dependent apparent anti-inflammatory effects associated with reduction of cytokines in the serum or brains. The dose of FK506 was chosen based on previously reported work that demonstrated protection against MPTP-induced dopamine depletion in the striatum of MPTP-injected C57BL/6 mice using 10 mg/kg/day FK506 [39]. The lack of protective effects of FK506 in MPTP-injected C57BL/6 mice was surprising. One possible explanation for this is that the MPTP injection paradigm is typically performed in male mice and our study was necessarily performed in female mice in order to be able to compare to the female humanized CD34+ mice. The fact that FK506 did not provide the benefits predicted may be due to sex differences. However, we cannot rule out the possibility that a different concentration of FK506 without overt toxicity may have also provided benefits in the female C57BL/6 mice. Due to limitations in the number of available humanized CD34+ mice for the study design, alternative FK506 dosage interventions were not possible. This increased toxicity susceptibility of C57BL/6 female mice to the dosage of FK506 used is, in itself, interesting and worth pursuing in future work. Understanding this vulnerability may give some insight into understanding the reason for drug-induced nephrotoxicity in some patients. Although we can only hypothesize, we expect that the loss of TH immunoreactivity in the C57BL/6 mice is primarily due to the more classic model of direct neurotoxicity by MPP^+^. Conversely, a significant portion of the loss of TH immunoreactivity in the CD34+ humanized mice involves an inflammatory component that is preventable by FK506 treatment.

Even though the specific changes in temporal phenotype of immune cells in both the brain and periphery during PD remain unclear, it is well-known that either of these cell types rely upon secretion of a number of cytokines, such as IL-1β, IL-6 and TNF-α to act via both paracrine and autocrine cellular mechanisms [15, 40]. In an effort to identify particular brain cytokines that were exclusive to the humanized immune system, we quantified an array of cytokines expression profiles in C57BL/6 mice vs. humanized CD34+ mice and serum cytokines vs. brain cytokines. We also compared the plasma cytokine profiles from human PD patients and healthy controls to identify a few cytokines such as IL-1α, IL-2, IL-4, and IL-6 as unique and robust serum biomarkers of human disease that were replicated in the brains and serum of the humanized CD34+ mice. This suggests opportunity for further studies involving immunosuppressive therapeutic targets and even enticing possibilities of small scale clinical trials using FK506 in order to quantify changes in serum levels of these cytokines to reflect an anti-inflammatory effect in the brain in correlation with behavioral improvement in PD patients.

Since the humanized CD34+ mice have not been very well characterized with regards to brain biology, it was difficult to know beforehand whether or not there would be any presence of human cells in the brains of 12 weeks post-engrafted mice. However, immunostaining against human specific macrophage antigens demonstrated the presence of sparse meningeal-like human CD45 and human HLA-DR specific immunoreactivity. The immunostaining was clearly not as robust in the brain as compared to the spleen and intestine, there is a possible role of parenchymal brain microgliosis and cytokine secretion in these mice. Nevertheless, we also cannot rule out the possibility that increases in human specific cytokines in both serum and brains of MPTP injected humanized CD34+ mice and detection of human macrophage markers such as CD68 and CD45 in peripheral organs such as intestine and spleen suggests that the peripheral immune system in these mice is effectively populated with human immune cells and that the cytokines produced by these cells may be infiltrating the brain from the periphery to perhaps drive the gliosis, behavioral deficits, and loss of TH staining. Indeed, recent work demonstrates the presence of lymphatic vessels in the brain that might allow for the cytokine or lymphocyte influx [41]. It would be very exciting to repeat this work with the humanized CD34+ mice and determine whether iv delivery of select human cytokines that were elevated by the MPTP treatment and in human PD plasma, IL-1α, IL-2, IL-4, or IL-6, are sufficient to produce the brain changes observed (i.e. gliosis, motor deficit, loss of TH staining, and elevated brain cytokines). Conversely, selective cytokine receptor antagonists or neutralizing antibodies may be able to attenuate the adverse effects of MPTP injection in these mice. This may provide significant insight into understanding whether a peripheral immune change can drive the brain disease in humans. Overall, this study demonstrates for the first time, the MPTP induced PD model in female humanized CD34+ mice and a possible therapeutic effect of FK506 in treating the MPTP-associated behavior deficits as well as inflammatory responses observed during the disease.

## Conclusions

These data demonstrate for the first time, induction of Parkinson’s disease-like symptoms in female humanized CD34+ mice using MPTP. There are distinct species differences between the cytokine profiles of humanized CD34+ mice and C57BL/6 mice. MPTP was able to produce loss of tyrosine hydroxylase immunoreactivity, loss of motor strength, and increase in proinflammatory cytokines in both strains of mice. However, these effects were attenuated using an immunosuppressant drug, FK506, only in the humanized CD34+ mice. Our data suggest that these mice may represent a more accurate model for assessing inflammatory changes in PD and developing anti-inflammatory therapeutics.

## Abbreviations

ANOVA: Analysis of Variance
CD: Cluster of Differentiation
EDTA: Ethylenediaminetetraacetic acid
ELISA: Enzyme-linked immunosorbent assay
GFAP: Glial fibrillary acidic protein
GM-CSF: Granulocyte macrophage colony-stimulating factor
HLA-DR: Human Leukocyte Antigen- antigen D Related
IFN: Interferon
IL: Interleukin
MPTP: 1-methyl-4-phenyl-1,2,3,6-tetrahydropyridine
MPTP-HCL: MPTP-hydrochloride
NFAT: Nuclear factor of activated T-cells
NSG: NOD scid gamma
OHDA: Hydroxydopamine
PBS: Phosphate-buffered saline
PD: Parkinson’s disease
SD: Standard deviation
TH: Tyrosine hydroxylase
TLA: Locomotor activity time
TNF: Tumor necrosis factor
